# Liquid Biopsy in Head and Neck Squamous Cell Carcinoma: A Molecular Perspective on Circulating Biomarkers and Their Clinical Translation

**DOI:** 10.3390/cimb48090853

**Published:** 2026-08-22

**Authors:** Francesca Cascone, Gabriele Riccardi, Dario Benelli, Riccardo Maurizi, Camilla Laureti, Carla Petrella, Carlo Cogoni, Antonio Minni, Christian Barbato

**Affiliations:** 1 Division of Otolaryngology-Head and Neck Surgery, Ospedale San Camillo de Lellis, ASL Rieti-Sapienza University, Viale Kennedy, 02100 Rieti, Italy; francesca.cascone@uniroma1.it (F.C.); gabriele.riccardi@uniroma1.it (G.R.); riccardo.maurizi1994@gmail.com (R.M.); antonio.minni@uniroma1.it (A.M.); 2 Department of Molecular Medicine, Sapienza University of Roma, Viale Regina Elena 324, 00161 Roma, Italy; carlo.cogoni@uniroma1.it; 3 Institute of Biochemistry and Cell Biology (IBBC), National Research Council (CNR), Viale del Policlinico 155, 00161 Roma, Italy; carla.petrella@cnr.it; 4 Interdisciplinary Department of Well-Being, Health and Environmental Sustainability (BeSSA), Sapienza University of Roma Via delle Fontanelle, 02100 Rieti, Italy

**Keywords:** head and neck squamous cell carcinoma, liquid biopsy, circulating tumor DNA, HPV, ctHPV DNA, minimal residual disease, microRNA

## Abstract

Liquid biopsy, the analysis of tumor-derived material in blood, saliva, and other body fluids, is increasingly explored for the diagnosis, surveillance, and molecular characterization of head and neck squamous cell carcinoma (HNSCC). Its performance, however, is not uniform across the disease, and the reason is fundamentally molecular. human papillomavirus (HPV)-positive oropharyngeal cancers carry viral oncogenes that are absent from the host genome and therefore provide a near ideal, tumor specific circulating marker, whereas HPV-negative tumors are driven by a heterogeneous somatic landscape that offers no single universal target. In this narrative review, we adopt a molecular perspective. We first examine the biological origin of circulating tumor DNA and of the other analytes that liquid biopsy can interrogate including circulating tumor HPV DNA, viral transcripts, microRNAs, extracellular vesicles, and methylation signatures. We then consider how analytical platforms, from droplet digital PCR to next generation and ultrasensitive whole-genome sequencing, translate these molecules into measurements. Only afterward do we discuss the clinical questions, organized by clinical objective rather than by individual study: diagnosis and early detection, prognosis and risk stratification, treatment response monitoring, minimal residual disease and surveillance, and biomarker guided de-escalation in HPV-positive disease. Twelve registered clinical trials, involving approximately 1183 patients, are presented as illustrations of these questions. We close on the biological and technical gaps that still separate promising signals from clinical practice, and on the multi analyte and dynamic strategies most likely to bridge them. At present, liquid biopsy should be regarded as a complementary tool rather than as a replacement for established clinicopathological assessment.

## 1. Introduction

Head and neck cancer is the sixth most common malignancy worldwide and remains a leading cause of cancer-related mortality [1]. Beyond its oncological burden, it affects quality of life profoundly, because of the anatomical and functional structures involved. The disease arises from two largely distinct routes. The first is the classical association with tobacco and alcohol, which produces predominantly human papillomavirus (HPV)-negative tumors of the oral cavity, larynx, and hypopharynx. The second is infection by high-risk human papillomavirus, mainly genotype 16, which underlies a growing fraction of oropharyngeal cancers (OPSCC). This distinction is not merely epidemiological. HPV-positive disease tends to respond better to chemoradiotherapy and to carry a more favorable prognosis, which has made it the focus of efforts to de-escalate treatment and spare long-term toxicity. These efforts, in turn, demand biomarkers able to identify who can safely receive less.

Current diagnosis relies on endoscopy, imaging, and tissue biopsy, while post-treatment follow-up still depends mainly on clinical examination and radiological imaging [2]. These approaches have intrinsic limits: imaging detects recurrence only once a lesion is macroscopically evident, and tissue sampling is invasive, captures a single site at a single time, and is poorly suited to repeated monitoring. Liquid biopsy addresses these gaps by interrogating tumor-derived material that circulates in blood, saliva, or other fluids, offering a minimally invasive and repeatable window on the disease [3]. Several classes of analyte have been investigated in this setting including circulating tumor cells (CTCs), circulating tumor DNA (ctDNA), microRNAs, and extracellular vesicles [4].

The aim of this review is to read these applications through a molecular lens. We argue that the behavior of liquid biopsy in neck squamous cell carcinoma (HNSCC), and especially the sharp difference between HPV-positive and HPV-negative disease, becomes intelligible only once the biology of the circulating analytes is made explicit. We therefore place that biology at the center, and let the clinical discussion and the registered trials that illustrate it, follow from it.

**Literature scope and methodology.** This narrative review includes a structured evaluation of trials formally registered in clinical databases. Peer-reviewed publications were retrieved from PubMed/MEDLINE and Embase by employing various combinations of the following keywords: liquid biopsy, circulating tumor DNA, circulating tumor HPV DNA (ctHPV DNA), tumor tissue modified viral DNA (TTMV DNA), microRNA, extracellular vesicles, head and neck squamous cell carcinoma, and oropharyngeal cancer. A separate search for actively registered clinical investigations was performed on ClinicalTrials.gov (15 April 2026) using the query “liquid biopsy head and neck”. Out of the 29 records obtained from this initial screening, 17 were removed from consideration, while the remaining 12 met the criteria and were incorporated in the present analysis.

Eligibility required that studies be formally recorded on ClinicalTrials.gov, focus exclusively on head and neck squamous cell carcinoma, assess a liquid-biopsy method, encompassing ctDNA, circulating tumor cells, exosomes, and additional blood or saliva-derived biomarkers, and adopt either an interventional or observational framework. Exclusion criteria comprised investigations into other neoplastic diseases, those covering various tumor types without distinct head and neck subgroup analyses, studies restricted to thyroid cancers, trials lacking a dedicated liquid biopsy evaluation, and reports with inadequate methodological descriptions. Given that the preliminary screening process was based solely on registry titles and brief study summaries, the twelve selected trials underwent subsequent verification regarding their registration status, primary objectives, and methodological features. This article is not intended as a systematic review or meta-analysis; instead, it aspires to deliver a synthesis grounded in molecular insights, rather than to compile an exhaustive inventory of all published evidence. Consequently, reliance upon a single clinical-trial database is acknowledged as a methodological limitation.

**Novelty of this review.** In contrast to previous reviews that largely presented the available evidence on a study by study basis, this article is structured around a unifying molecular concept: the performance of liquid biopsy in HNSCC is strongly influenced by the biological nature of the analyte being detected. In HPV-positive disease, viral oncogenes represent tumor-associated sequences that are absent from the host genome and therefore offer highly specific molecular targets, whereas HPV-negative tumors are characterized by a heterogeneous spectrum of somatic alterations without a comparable universal marker. This molecular framework is complemented by a clear distinction between analytical validity, clinical validity, and clinical utility, and by an organization of the available evidence according to the clinical question addressed rather than the individual study.

## 2. Molecular Basis of Liquid Biopsy in HNSCC

### 2.1. Biological Origin of Circulating Tumor DNA

Cell-free DNA circulates in the blood of healthy individuals, released mainly through apoptosis and necrosis, and to a lesser extent, through active secretion. In patients with cancer, a variable fraction of this pool, the circulating tumor DNA, originates from tumor cells and carries their genetic and epigenetic alterations. Because cell-free DNA has a short half-life, on the order of minutes to a few hours, its abundance reflects the tumor in close to real-time, which is what makes serial sampling informative [5]. The fragmentation of cell-free DNA is itself non-random: fragments cluster around the length of nucleosomal units, and tumor-derived molecules show distinctive size and end motif patterns. This fragmentomic signal is increasingly exploited to distinguish the tumor from the background even when the underlying mutations are unknown.

### 2.2. ctHPV DNA as a Privileged Target in HPV-Positive Disease

The single most important molecular fact for liquid biopsy in HNSCC is that high-risk HPV oncogenes are foreign to the human genome. In HPV-positive OPSCC, fragments of viral DNA, often derived from integrated virus, are shed into the circulation as so-called tumor tissue modified viral (TTMV) DNA. Since viral sequences are absent from the host’s germline DNA, ctHPV DNA offers a level of specificity generally unattainable with standard somatic tumor markers [6]. However, false-positive results remain a possibility. Transient or recent oral HPV infections, detection of HPV-derived DNA from benign lesions, genotype panels that do not fully capture high-risk types beyond HPV16, and dependence on a single time point measurement can all yield weak positive signals unrelated to the actual malignancy. Consequently, confidently attributing a circulating signal to cancer requires broad panels covering high-risk HPV genotypes, the identification of viral integration signatures characteristic of tumor tissue, and longitudinal monitoring of biomarker trends, rather than interpreting an isolated positive result. These molecular features help clarify why the most mature liquid biopsy applications in HNSCC have thus far been developed largely for HPV-driven disease [7]. The same principle underlies the use of ctHPV DNA both as a quantitative measure of tumor burden and as a sensitive sentinel of residual or recurrent disease.

### 2.3. The Somatic Landscape of HPV-Negative HNSCC

HPV-negative tumors offer no equivalent of a foreign viral marker. Their genomes are dominated by alterations in TP53, the most frequently mutated gene in this disease, together with CDKN2A loss and a recurrent but heterogeneous set of other events [8]. Two consequences follow for liquid biopsy. First, detection generally requires either a tumor-informed panel, built from each patient’s own mutations, or a sufficiently broad untargeted panel, both of which are more complex and more costly than a single viral assay. Second, the very mutations that mark the tumor, TP53 above all, also accumulate in aging and inflamed mucosa and in clonal hematopoiesis, which raises the background and complicates interpretation. Together with the often lower shedding of these tumors, this makes minimal residual disease detection intrinsically harder in HPV-negative than in HPV-positive HNSCC, and it is the main reason the field has advanced unevenly across the two subsets [9].

Several complementary approaches have been developed to improve detection in the more challenging setting of HPV-negative disease. Sequencing of matched leukocytes or white blood cells can help distinguish tumor-derived alterations from clonal hematopoiesis, an important source of background signal for TP53 and other genes that may also be mutated in age-related hematopoietic clones. Tumor-informed personalized panels, designed around the mutations identified in an individual patient’s tumor, can increase sensitivity at the patient level, although they depend on the availability of tumor tissue and require tailored bioinformatic analysis. In contrast, fixed tumor-agnostic panels are easier to implement but usually provide lower sensitivity. Methylation based assays add a mutation-independent source of information, whereas fragmentomic methods take advantage of differences in fragment length and end-motif patterns between tumor-derived and non-tumor DNA. The extent of biomarker shedding also varies according to anatomical site: saliva and oral-rinse samples tend to be more informative for tumors of the oral cavity and oropharynx than for laryngeal or hypopharyngeal cancers. These differences support selecting the biofluid according to the location of the primary tumor.

### 2.4. Analytes Beyond Circulating DNA

DNA is the most developed analyte, but not the only one. Viral transcripts, in particular E6 and E7 RNA, report on transcriptional activity rather than mere presence of the virus. Circulating microRNAs are a second avenue: miR-375, for instance, is consistently downregulated in HNSCC and its low expression is associated with worse outcome, which has made it a candidate prognostic and circulating biomarker [10,11], even though the available clinical evidence remains limited and largely HPV independent. miR-375 is considered here as one of several circulating microRNAs with potential prognostic relevance. Although its regulation has been mechanistically linked to HPV E6/E7 activity [12], this aspect lies outside the main scope of the present review.

Extracellular vesicles, including exosomes, carry nucleic acids and proteins that may stabilize otherwise labile signals and report on tumor signaling [13,14]. Aberrant DNA methylation provides a further layer, often detectable independently of specific mutations [15]. Finally, the choice of biofluid matters: saliva and oral rinses are particularly attractive for tumors of the oral cavity and oropharynx, where local shedding can exceed that seen in plasma, and combining fluids may improve sensitivity [16,17]; even exhaled breath has been investigated as a source of volatile tumor-associated compounds [18]. Table 1 summarizes how clinical category, main analyte, platform, and underlying biology relate.

CTCs form another part of the circulating biomarker pool. In HNSCC, various studies have demonstrated connections between CTC burden and patient prognosis, where increased cell numbers are linked to nodal spread and a worse clinical outlook. Nevertheless, widespread adoption into routine care is hindered by their scarce concentration in blood, dependence on the specific enrichment approach employed, and the absence of fully harmonized analytical protocols. For now, CTCs thus appear better suited as a supplementary prognostic tool rather than as a standard clinical assay applied on a regular basis.

## 3. Analytical Platforms and Methods

The molecules described above become clinically useful only through assays able to detect them at very low abundance. Droplet digital PCR (ddPCR) offers exquisite sensitivity for a small number of predefined targets and is well-suited to viral sequences, which is why it has emerged as a leading approach for ctHPV DNA in HPV-positive disease [19]. Its limitation is multiplexing, since it interrogates only the targets it is designed for, and it is therefore best suited to the longitudinal monitoring of known markers. Next generation sequencing (NGS), in contrast, can survey many alterations at once, through tumor-informed panels tailored to each patient or through fixed broad panels, at the cost of greater complexity, and often lower sensitivity per target [20]. The two are best regarded as complementary rather than mutually exclusive. More recent approaches combine hybrid capture, whole genome sequencing and fragmentomics to screen many HPV genotypes and to integrate viral reads with fragmentation features, reporting very high sensitivity and specificity in case–control settings [21]. Across all platforms, the recurring issues are the limit of detection, the absence of standardized units and thresholds, and pre-analytical variability, all of which determine whether a given molecular signal can support a clinical decision.

## 4. Clinical Applications

Organizing the clinical discussion by question, rather than by individual study, makes the strengths and weaknesses of liquid biopsy easier to weigh.

Throughout this section, the available evidence is considered at three distinct levels. Analytical validity refers to the reliable and reproducible measurement of the analyte, whereas clinical validity describes the relationship between that measurement and a defined clinical condition or outcome, including its prognostic significance. Clinical utility, in contrast, requires evidence that using the biomarker to guide clinical management results in improved patient outcomes. In HPV-positive disease, ctHPV DNA is supported by an expanding body of evidence for both analytical and clinical validity, while its clinical utility still requires confirmation in prospective interventional studies. Table 2 summarizes the characteristics of the registered clinical trials that provide the interventional background to this discussion. Table 3 summarizes the level of evidence readiness across the main clinical applications, whereas Table 4 provides a structured comparison of representative studies together with their reported performance metrics.

### 4.1. Diagnosis and Early Detection

In HPV-positive disease, ctHPV DNA generally demonstrates a high degree of concordance with tissue-based HPV testing, although performance varies depending on the assay used, suggesting that liquid biopsy may serve as a non-invasive surrogate for HPV-driven OPSCC [22]. This is particularly relevant given the evidence of p16 and HPV discordance in European cohorts, where p16 positivity alone may overestimate the true prevalence of HPV-driven disease and thereby distort prognostic stratification and treatment decisions [23,24]. Liquid biopsy may also mitigate limits of conventional sampling, since HPV-positive OPSCC frequently presents with cystic cervical metastases that reduce the accuracy of fine needle aspiration cytology [25]. The main caveat for screening is statistical rather than analytical: in low prevalence populations, even a highly specific test generates false positives, so positive predictive value must be evaluated before adoption.

### 4.2. Prognosis and Risk Stratification

Baseline levels of ctHPV DNA broadly track tumor burden and stage, and both the initial level and the rate of clearance during treatment carry prognostic information. This positions liquid biopsy as a complement to anatomical staging, refining the identification of patients at higher risk of relapse, and potentially, those suitable for de-escalation.

### 4.3. Treatment Response Monitoring

Because circulating tumor markers turn over rapidly, their dynamics during chemoradiotherapy can report on response sooner than imaging. This matters especially because post-treatment PET-CT carries a substantial rate of false-positive findings after chemoradiotherapy in HPV-positive disease, exposing patients to repeated imaging and invasive procedures [26,27,28]. Rapid clearance of ctHPV DNA is generally associated with favorable outcome, whereas persistence flags inadequate response and an early opportunity to reconsider management.

### 4.4. Minimal Residual Disease and Surveillance

This is the application where liquid biopsy is closest to clinical utility [29]. After curative intent treatment, serial measurement of ctHPV DNA can reveal occult residual or recurrent disease; in several reports, weeks to months before clinical or radiological detection. Post-treatment plasma and saliva HPV DNA testing identified recurrence earlier than standard imaging based follow-up [30], and persistently undetectable ctHPV DNA was associated with the absence of recurrence, whereas rising levels correlated with progression [31]. The high negative predictive value of a negative result is what makes surveillance in HPV-positive OPSCC the setting in which these assays have moved the fastest toward routine use.

### 4.5. Treatment De-Escalation in HPV-Positive OPSCC

The favorable biology of HPV-positive disease has driven interest in reducing treatment intensity to limit long-term toxicity, but doing so safely requires the reliable identification of low-risk patients. Trials such as AVOID and ECOG-ACRIN E3311 have shown that selected HPV-positive patients may receive less intensive adjuvant treatment without compromising outcomes [32,33]. By quantifying residual molecular disease, ctHPV DNA-based assessment is an attractive candidate to stratify patients within such strategies, and this is among the most actively investigated questions in the field. It must be stated clearly, however, that current evidence does not yet justify omitting indicated adjuvant treatment based on a negative result, nor escalating treatment based on persistent positivity, in the absence of prospective validation.

Before ctHPV DNA can be implemented into escalation or de-escalation treatment strategies, several prerequisites must still be satisfied. First, decision cut-offs should be defined and confirmed prospectively, rather than obtained through retrospective post hoc evaluations. Second, explicit protocols for longitudinal testing are required, including mandatory confirmation by two successive positive samples or the application of predetermined clearance and kinetic rules, so as to minimize the impact of sporadic false-positive readings. Third, biomarker-guided management algorithms must clarify how a molecular observation should translate into specific treatment adjustments, and subsequently prove their benefit in prospective interventional trials. Pending the availability of such evidence, ctHPV DNA results may aid clinical decision-making and support intensified surveillance, but should not replace the established indications for adjuvant treatment.

## 5. The Clinical Trial Landscape

Rather than catalogue individual studies, it is more informative to map the questions that current trials are addressing. A search of ClinicalTrials.gov identified twelve registered trials investigating liquid biopsy in HNSCC, enrolling approximately 1183 patients in total, which cluster around a small number of clinical objectives and are best read as illustrations of the questions above. Their characteristics are summarized in Table 2.

Several ongoing and recently registered studies focus on surveillance and the detection of minimal residual disease. PRE-MERIDIAN (NCT04599309) investigates real-time monitoring of ctDNA and ctHPV DNA in patients with locally advanced HNSCC treated with curative intent, while a Danish study (NCT06592716) examines whether recurrence of oropharyngeal cancer can be identified through blood-based testing. Clear-HPVca (NCT06730412) assessed an ultrasensitive whole-genome HPV sequencing approach for the detection of molecular residual disease. In HPV-negative oropharyngeal cancer, one interventional study integrates tumor mutational burden, liquid-biopsy analysis, and DCE-MRI perfusion imaging (NCT07407205).

Other trials are centered on molecular and immune profiling. These include the evaluation of PD-L1 and additional biomarkers on circulating tumor cells (NCT04490564), the analysis of ctDNA kinetics during immuno-oncology treatment in the IO-KIN study (NCT04606940), and the longitudinal assessment of HPV DNA in liquid-biopsy samples (NCT05774561). A further group of recruiting studies broadens the application of liquid biopsy toward diagnosis and the use of alternative biofluids. Examples include the ENHANCE study for early diagnosis (NCT05645783), a multicenter Italian translational study combining plasma, saliva, and tissue analyses with ctHPV DNA, microRNA, and NGS profiling (NCT05918510), a microfluidic platform based on natural killer cells (NCT06678724), and prospective cohorts collecting both blood and saliva specimens (NCT05122507 and NCT03926468). Considered together, these studies indicate that the field is increasingly converging on HPV-positive disease for surveillance applications, while simultaneously exploring more challenging settings such as HPV-negative tumors and multi-analyte detection strategies.

Table 5 reports the registry details of these trials, including sponsor country, study design, condensed primary endpoint, target enrollment, and the date of the most recent registry update.

## 6. Challenges, Limitations and Standardization

Several obstacles still separate promising signals from clinical practice. Biologically, HPV-negative tumors lack a universal marker and may shed little DNA, and sensitivity remains limited in early stage and low burden residual disease. Technically, the field suffers from heterogeneous limits of detection, an absence of standardized units and thresholds, and pre-analytical variability that hampers comparison across studies. Clinically, most evidence is still observational and derived from small, heterogeneous populations, and the decisive demonstration, that acting on a liquid biopsy result actually improves patient outcomes, requires prospective interventional trials that are only now maturing. A specific methodological caution applies to reviews built on a single registry: reliance on ClinicalTrials.gov alone may underrepresent non-U.S. or non-registered protocols. Cost, access, and regulatory validation add further constraints. Recognizing these limits is not a counsel of pessimism, but a map of what must be resolved.

## 7. Future Perspectives

The most likely path forward is integrative. Combining analytes, for example, viral DNA with fragmentomic and methylation features, or DNA with microRNAs and extracellular vesicle cargo, may extend sensitivity into the settings where single markers fail including HPV-negative disease and minimal residual disease. Fragmentomics and methylation profiling are promising precisely because they do not depend on knowing the tumor’s mutations in advance. The microRNA pathway could merit further clinical exploration as a possible origin of circulating biomarkers. Specifically, the functional connection between miR-375 and the regulation of viral oncogenes, along with its potential role in therapy-induced senescence within HPV-positive cells, currently remains only an experimental hypothesis. Since this idea has not yet been corroborated by published liquid-biopsy outcome data, it is cited here merely as a potential direction for future research. Pairing liquid biopsy with imaging, and embedding it in prospective surveillance and de-escalation trials with harmonized assays, will be necessary to convert biological plausibility into standard of care.

## 8. Conclusions

Liquid biopsy in HNSCC is best understood from its molecular foundations. The foreign nature of HPV oncogenes gives ctHPV DNA a specificity that has carried the HPV-positive setting, and especially minimal residual disease surveillance, to the threshold of clinical utility, while the heterogeneous somatic landscape of HPV-negative disease explains why progress there has been slower. A molecular reading therefore does more than organize the evidence: it predicts where liquid biopsy will work, where it will struggle, and which multi-analyte and dynamic strategies are most likely to close the remaining gap between a measurable signal and a clinical decision. For now, liquid biopsy should be regarded as a complementary diagnostic and prognostic tool, pending the prospective validation that will determine its place in routine management.

## Figures and Tables

**Table 1:** Molecular reading of liquid biopsy applications in HNSCC.

Clinical Category	Main Analyte	Molecular Platform	Biological Thread
Diagnosis and early detection	ctHPV DNA, E6/E7 transcripts	ddPCR, ultrasensitive HPV whole-genome sequencing	Viral oncogene sequences are absent from the host genome
MRD and surveillance	ctHPV DNA, TTMV DNA	ddPCR, HPV whole-genome sequencing	Tumor specific viral fragments act as a sentinel of residual disease
Treatment response prediction	ctDNA and ctHPV DNA dynamics, PD-L1, TMB	ddPCR, NGS	Rapid turnover of tumor-derived molecules reports response in near real-time
Molecular profiling	Somatic ctDNA, methylation, miRNA, extracellular vesicles	NGS, methylation assays	Heterogeneous somatic landscape and analytes beyond DNA

ctHPV DNA, circulating tumor HPV DNA; TTMV, tumor tissue modified viral DNA; ctDNA, circulating tumor DNA; miRNA, microRNA; ddPCR, droplet digital PCR; NGS, next generation sequencing; TMB, tumor mutational burden; PD-L1, programmed death-ligand 1; MRD, minimal residual disease.

**Table 2:** Main characteristics of the registered clinical trials investigating liquid biopsy in HNSCC (twelve trials, approximately 1183 patients).

Title	Study Type	Status	N	Analyte Detected	Tumor Type	Method	Start	Last Update	Planned End
Liquid biopsy for early diagnosis of HNSCC (ENHANCE)	Observational	Recruiting	170	Blood (ctDNA)	HPV-related HNSCC (stage I, II)	NGS	01/02/23	09/08/24	12/2024
Role of liquid biopsies in HPV-associated cancer treatment monitoring	Observational	Recruiting	200 (Arm A)	Blood (ctDNA), gargle washes, swabs, exhaled breath condensate	Oropharyngeal, HPV-positive	ddPCR	01/06/22	12/02/26	01/12/26
Viral biomarkers and microRNAs in oropharyngeal and occult HPV tumors	Observational	Recruiting	142	Blood (ctDNA, HPV DNA, miRNA)	Oropharyngeal, HPV-positive, and occult HPV related	Not stated, PDX	04/04/22	10/10/23	04/04/29
Microfluidic liquid biopsy platform for recurrent/metastatic HNSCC using NK cell IFN-gamma	Observational	Recruiting	30	Blood (circulating micrometastases, NK cells, IFN-gamma)	Oral cavity, oropharynx, hypopharynx, larynx	Other	01/01/25	24/02/26	31/12/27
Real-time ctDNA and HPV DNA in high risk locally advanced HNSCC (PRE-MERIDIAN)	Observational	Active, not recruiting	35	Blood (ctDNA, HPV DNA)	Locally advanced HNSCC (III HPV+, III–IV HPV-)	NGS, ddPCR	15/10/20	10/06/26	30/06/26
TMB, liquid biopsy, angiogenic factors and DCE-MRI in HPV-negative oropharyngeal cancer	Interventional	Active, not recruiting	30	Blood (ctDNA) and DCE-MRI	HPV-negative, oropharyngeal	Not stated	10/06/19	17/02/26	31/12/26
Can recurrence in the oropharynx be detected by blood samples?	Observational	Active, not recruiting	200	Blood (ctDNA, HPV DNA)	Oropharyngeal, HPV-positive	ddPCR	02/04/24	18/11/25	31/12/28
ctDNA for residual disease in HPV-associated HNSCC (Clear-HPVca)	Observational	Completed	103	Blood (ctDNA)	HPV-related HNSCC (stage I–III)	HPV-DeepSeek (NGS)	01/08/20	04/12/25	31/10/24
Validation of molecular diagnostic assays (HNSCC, NSCLC, melanoma)	Observational	Completed	25	Blood (PD-L1) in CTC	HNSCC, recurrent and metastatic, post immunotherapy	Other	25/06/19	30/12/25	07/09/22
ctDNA kinetics in immuno-oncology (IO-KIN)	Observational	Completed	18	Blood (ctDNA)	HNSCC, recurrent and metastatic, post immunotherapy	Not stated	19/10/20	19/05/22	12/10/21
Liquid biopsy in head and neck cancer	Observational	Unknown	30	Blood (ctDNA)	HNSCC after chemo/radiotherapy	ddPCR	01/08/19	04/06/19	31/12/22
Liquid biopsy of head and neck cancer patients in blood and saliva	Observational	Unknown	200	Blood (ctDNA)	HNSCC after chemo/radiotherapy	NGS, ddPCR	08/05/17	12/04/23	04/2023

ctDNA, circulating tumor DNA; HPV DNA, circulating tumor HPV DNA; ddPCR, droplet digital PCR; NGS, next generation sequencing; CTC, circulating tumor cell; TMB, tumor mutational burden; PDX, patient-derived xenograft; DCE-MRI, dynamic contrast enhanced MRI, NK, natural killer; IFN-gamma, interferon gamma; NSCLC, non-small cell lung cancer. Trial two reports the Arm A enrolment. Dates are shown in the Day Month Year format, or as Month Year where only month precision is registered.

**Table 3:** **Evidence-readiness map for liquid biopsy across major clinical applications in HNSCC.** Evidence strength and clinical readiness were qualitatively assessed on the basis of the studies discussed in this review, with readiness classified as investigational, emerging, or near-ready.

Clinical Question	Principal Analyte	HPV+ Evidence	HPV+ Readiness	HPV- Evidence	HPV- Readiness
Diagnosis and early detection	ctHPV DNA; somatic ctDNA	Moderate	Emerging	Low	Investigational
Prognosis and risk stratification	ctHPV DNA load and clearance	Moderate	Emerging	Low	Investigational
Treatment response monitoring	ctHPV DNA kinetics	Moderate	Emerging	Low	Investigational
MRD and surveillance	ctHPV DNA, TTMV DNA	High (prognostic)	Near-ready	Low to moderate	Investigational
Treatment de-escalation	ctHPV DNA	Low (utility unproven)	Investigational	Not established	Investigational

**Table 4:** Structured comparison of representative diagnostic and surveillance performance from syntheses cited in this review. Each row reports pooled or single-cohort figures with 95% confidence intervals and states its source; the table can be extended with additional per-study rows.

Study (Source)	Analyte/Biofluid/Platform	Setting (HPV Status, Stage)	Sensitivity (95% CI)	Specificity (95% CI)	PPV/NPV or LR	Notes
Campo et al., 2024, meta-analysis [6]	ctHPV DNA and TTMV DNA; plasma; ddPCR	HPV+ OPSCC; post-treatment surveillance	86% (78–91)	96% (91–99)	PLR 24.7; NLR 0.072	12 studies, 1311 patients; AUC 0.81 (0.67–0.91); may detect recurrence before imaging
Campo et al., 2024, cohort [22]	ctHPV DNA; plasma; ddPCR (E6 HPV16/33/35)	HPV+ OPSCC; follow-up	100% (72.1–100)	98.4% (91.7–100)	PPV 90.9 (62.3–98.4); NPV 100 (94.3–100)	Single-cohort follow-up performance
Adeoye et al., 2022, meta-analysis [15]	Hypermethylated DNA panels; saliva and oral swabs	OSCC; diagnosis	86.2% (60–96.2)	90.6% (85.9–93.9)	PLR 9.2; NLR 0.15	11 studies; panels (individual markers lower, 70%); high QUADAS-2 bias concerns
Naegele et al., 2024, meta-analysis [19]	ctHPV DNA; plasma; qPCR vs. ddPCR vs. NGS	HPV+ cancers incl. OPSCC; diagnosis	qPCR 0.51 (0.37–0.64); ddPCR 0.81 (0.73–0.87); NGS 0.94 (0.88–0.97)	qPCR 0.93 (0.83–0.97); ddPCR 0.98 (0.96–0.99); NGS 0.95 (0.90–0.97)	NR	36 studies, 2986 patients; pooled across HPV-associated cancers; NGS most sensitive, specificity similar

CI, confidence interval; PPV, positive predictive value; NPV, negative predictive value; LR, likelihood ratio; PLR, positive likelihood ratio; NLR, negative likelihood ratio; AUC, area under the curve; OSCC, oral squamous cell carcinoma; QUADAS-2, Quality Assessment of Diagnostic Accuracy Studies, version 2; NR, not reported.

**Table 5:** **Registry audit of the clinical trials summarized in Table 2.** Registry identifier, sponsor country, study design, condensed primary endpoint, target enrollment, and date of the most recent registry update were obtained from ClinicalTrials.gov using the search query “liquid biopsy head and neck”. The overall figure of approximately 1183 participants represents the combined planned enrollment across the included studies.

Trial	Registry ID	Country	Phase/Design	Primary Endpoint	Target Enrollment	Last Update Posted
ENHANCE	NCT05645783	UK	Observational	Sensitivity of NGS ctDNA assay in early HNSCC (1 y)	170	09/08/24
HPV treatment-monitoring liquid biopsy	NCT05774561	Czech Republic	Observational	ctHPV DNA and HPV status dynamics in liquid biopsy; correlation with prognosis and TMB (4 y)	200 (Arm A)	12/02/26
Viral biomarkers and microRNAs	NCT05918510	Italy	Observational	Accuracy; HPV-DNA and miRNA kinetics in oropharyngeal and occult primary tumors (baseline)	142	10/10/23
Microfluidic NK IFN-gamma platform	NCT06678724	Taiwan	Observational	Progression-free survival (up to 60 mo)	30	24/02/26
PRE-MERIDIAN	NCT04599309	Canada	Observational	Real-time detection of ctDNA and/or HPV DNA (CAPP-Seq, dPCR) in high-risk LA-HNSCC (up to 2 y)	35	10/06/26
TMB and DCE-MRI (HPV negative)	NCT07407205	Slovenia	Interventional	Predictive value of liquid-biopsy biomarkers and DCE-MRI for chemoradiotherapy response (up to 6 mo)	30	17/02/26
Recurrence detection by blood	NCT06592716	Denmark	Observational	Sensitivity and specificity of ddPCR ctHPV-DNA vs. verified recurrence (up to 3 y)	200	18/11/25
Clear-HPVca	NCT06730412	USA	Observational	Disease-free and overall survival by post-surgical MRD detection (2 y)	103	04/12/25
Validation of molecular diagnostic assays	NCT04490564	Greece	Observational	Clinical performance (sensitivity, specificity, PPV/NPV) of PD-L1 kit in CTCs and tissue (12 mo)	25	30/12/25
IO-KIN	NCT04606940	Canada	Observational	Change in ctDNA kinetics on immune checkpoint inhibitors (up to 1.5 y)	18	19/05/22
Liquid biopsy in head and neck cancer	NCT03926468	Finland	Observational	Diagnostic performance of ctDNA by ddPCR at baseline and 3-month follow-up	30	04/06/19
Blood and saliva	NCT05122507	Germany	Observational	Early recurrence detection lead time, liquid biopsy vs. clinical recurrence (up to 24 mo)	200	12/04/23

## Data Availability

No new data were created or analyzed in this study. Data sharing is not applicable to this article.
